# Dr. Daniel Hennessey

**Published:** 1917-01

**Authors:** 


**Dr. Daniel Hennessey,** Geneva 1866, of Bangor, Me., died last month aged 79. He was a member of the surgical staff of the Aide-de-Camp Hospital on David's Island, N. Y., during the Civil War.

